# Greater parental health literacy does not improve assessment of their child’s emergency condition severity

**DOI:** 10.1186/s12873-026-01541-8

**Published:** 2026-03-17

**Authors:** Eva-Maria Steinke, Till Milde, Hans Proquitté, Birgitta Hucke, Melanie Rohmann, Stefan Wolke, Thomas Lehmann, Luana Vogler-Dos-Santos, Jan-Christoph Lewejohann, Matthias Nuernberger

**Affiliations:** 1 Department of Emergency Medicine, Jena University Hospital, Friedrich Schiller University, Am Klinikum 1, 07747 Jena, Germany; 2 Department of Medical Statistics, Informatics and Data Science, Jena University Hospital, Friedrich Schiller University, Jena, Germany; 3 Department of Pediatrics and Adolescent Medicine, Jena University Hospital, Friedrich Schiller University, Jena, Germany; 4 Department of Pediatric Surgery, Jena University Hospital, Friedrich Schiller University, Jena, Germany; 5 Comprehensive Cancer Center Central Germany (CCCG), Jena, Germany; 6 Hopp Children’s Cancer Center Heidelberg (KiTZ), Heidelberg, Germany; 7 Clinical Cooperation Unit Pediatric Oncology, German Cancer Research Center Heidelberg (DKFZ), Heidelberg, Germany

**Keywords:** Pediatric emergency medicine, Emergency medicine, Health literacy, Emergency medicine, Emergency condition severity, Public health, Health seeking behavior

## Abstract

**Introduction:**

Low health literacy (HL) can impair decision-making in emergencies. Extending our preceding adult study, this preregistered observational study examined whether parental HL affects accuracy of severity assessments in the pediatric emergency department (PED) and whether the validity of children’s self-reports is age dependent.

**Methods:**

This single-center prospective cross-sectional study enrolled 182 pediatric patients and parents presenting to a German university hospital PED. Subjective and independent emergency condition severity assessments were obtained from patients, parents, and medical staff. Parental HL was measured using the 16-item European Health Literacy Survey questionnaire (HLS-EU-Q16). Reference standard severity assessment was retrospectively collected by specialists (retrospective chart-based chase assessment). Analyses compared assessment accuracy across three parental HL level groups (adequate, problematic, inadequate). Assessments were adjusted for covariates except for regression analyses, which we performed using unadjusted values.

**Results:**

Parental HL showed no measurable effect on assessment accuracy, discrepancy or concordance with medical team’s evaluations. Children’s self-reported anxiety emerged as a stronger predictor of actual clinical severity than HL measurements, with this relationship becoming significant at 8 years of age. Children’s severity assessment (OR = 1.38 [1.20, 1.57], *p* < .001) and less distinctly anxiety (OR = 1.15, 95% CI [1.01, 1.31], *p* = .037) predicted severe outcome, while the interaction between age and anxiety (B = 0.071, SE = 0.031, *p* = .022) showed that anxiety becomes a reliable indicator at around early school age. Concordance with the team’s assessment likewise emerged at school age (ρ ≈ 0.30, *p* < .05). Direct children’s self-assessment provided clinically meaningful information across all HL groups. Nursing assessments remained the strongest predictor of severe outcomes (OR = 1.95, 95% CI [1.37, 2.67], *p* < .001), followed by physicians (OR = 1.62, 95% CI [1.20, 2.19], *p* < .001). Parents were in our study not able to predict severe outcome (*p* = .22). Parental underestimation relative to specialists was consistently linked to severe outcomes (26–33%), regardless of HL level.

**Conclusion:**

Contrary to common assumptions, parental HL neither impaired nor improved recognition of pediatric emergency severity, and HL did not systematically shift discrepancy patterns. Instead, developmental maturity and professional observation are relevant for severity assessment. From early school age (7–8 years), children’s anxiety appears to provide an increasingly valid, dose-responsive signal of severity. Nurses’ assessments provided the highest predictive accuracy, underscoring their central role in triage. Age-appropriate use of pediatric patients’ self-assessments could further support risk stratification in the PED.

**Clinical trials registration:**

This observational study was preregistered in the German Clinical Trials Register (DRKS): DRKS00034108, Registration Date: 30th January 2025.

**Supplementary Information:**

The online version contains supplementary material available at 10.1186/s12873-026-01541-8.

## Introduction

Pediatric emergency departments (PED) face challenges that differ from, yet parallel, those in adult emergency medicine. In most cases, children present with parental accompaniment, and the pathway to emergency care depends on how both child and parent assess the severity of the situation with this judgment fundamentally influenced by health literacy (HL) [1]. More than half of adults in the United States and Europe have limited HL [2, 3], which can lead to both overestimation and underestimation of symptom severity [4] while it affects decisions about seeking PED care [5, 6]. Health literacy is defined as the ability to understand and use health information and is typically assessed through standardized questionnaires. Low HL is associated with poor health outcomes, reduced healthcare access, and lower educational and socioeconomic status [2, 7–9]. For parents, low HL can impair decision-making for their children, resulting in unnecessary PED visits that strain emergency resources or, conversely, delayed presentations that place the child at risk [5, 10]. Since pediatric and adolescent HL must largely be inferred from parental HL due to the lack of validated pediatric assessment tools [11], there remains limited understanding of children’s actual health-related knowledge. Especially, as there is evidence for non-concordance between child and proxy regarding situational assessments [12, 13]. Food literacy (FL) – a comprehensive subunit of HL – might serve as a surrogate parameter.

Evaluating the severity of an emergency is inherently subjective and shaped by factors such as HL, resulting in differing perceptions of urgency between patients and healthcare professionals.

Previous research has shown that parental HL and anxiety about child health can lead to discrepancies between parent perception and clinician assessment, yet the results are not unambiguous [14]. This study examined the relationship between parental HL, children’s self-assessment of emergency severity, parental assessment, and evaluation by medical professionals in a high-volume pediatric emergency setting. The study aimed to examine whether and in what ways parental HL relates to differences in severity assessments among children, parents, and emergency medicine professionals.

## Research questions

This study aimed to investigate two central questions regarding communication and clinical assessment in pediatric emergency care. First, we examined whether parental HL influences the accuracy of parents’ or patients’ evaluations of illness severity:

Does low parental HL impair the ability to recognize the severity of a child’s emergency condition? Does low HL increase the discrepancy between parental or child self-assessments and professional medical judgments? Does higher HL reduce such discrepancies?

Second, we explored the age-dependent validity of patients’ self-reports:

How does pediatric patients’ age affect the predictive value of self-rated illness severity, pain, and anxiety for clinically severe outcomes? How does the predictive value of child-reported severity, pain, and anxiety change across developmental stages, and from approximately which age onward do children’s self-assessments begin to show meaningful alignment with professional evaluations? Can a child’s FL predict their parents’ HL and serve as a surrogate for HL?

## Methods

### Study design and population

This study was a single-center, prospective, quantitative, analytical, cross-sectional observational investigation carried out in the PED of a German university hospital. The methodology largely corresponds to that used in the previous study in the adult ED [15]. The two emergency departments are separate in terms of space, patients, and personnel, with no overlap between them.

The aim of this study was to examine the influence of HL on the subjective assessment of emergency condition severity. The patient population consisted of PED cases who presented themselves with a parent during the day. The study was conducted between March 10, 2025, and June 10, 2025. Written informed consent was obtained from all participants. The analytical cross-sectional design adhered to the Strengthening the Reporting of Observational Studies in Epidemiology (STROBE) guidelines [16]. A pilot study was conducted prior to the main investigation to assess feasibility (*n* = 20). Ethical approval was granted by the Ethics Committee of Friedrich-Schiller-University Jena, Germany (Registration number: 2024-3336-Bef; date: May 7, 2024), and the study was preregistered on drks.de (DRKS00034108). All procedures followed relevant guidelines, regulations, and the principles of the Declaration of Helsinki II.

Participants were eligible for inclusion if accompanied by at least one parent or legal guardian. Patients admitted via emergency medical services (EMS) were excluded because it was no longer possible to obtain an unbiased assessment from the medical team after the handover. Admission by EMS is rare in the PED (on average 1.5 per day) Children who were critically ill and in obvious need of immediate treatment were not included in order to avoid delaying treatment under any circumstances. Such non-inclusions were very rare (*n* = 2). Sampling was performed daily during study hours, which rotated randomly among five time slots: 10–12 AM, 12 AM–2 PM, 2–4 PM, 4–6 PM, and 6–8 PM (see Supplementary 1 for details). Patients were recruited consecutively. The PED records approximately 10,000 visits annually.

### Administration and study procedure

Study procedure was adapted from the preceding study [15] for use in the PED. Differences in the present study are the inclusion of parents and pediatric patients who, due to their inability to read, rely on pictorial representations or who are not yet able to complete complex questionnaires without assistance.

Upon admission and completion of the initial ESI triage, patients and their parents were approached by the study team, including the principal investigator, who informed them about the study and invited their participation. After obtaining written informed consent from the parents, both patients and parents were asked to complete questionnaires assessing their subjective perception of the emergency condition’s severity. The questionnaires were completed privately in a quiet, separate room. Parents and children were seated a few meters apart, within sight of each other but without communication during completion. Patients received assistance from a member of the study team, while parents filled out their questionnaires independently. Completion took approximately six minutes (range: 4–9 min). If a medical necessity arose during data collection, the process was immediately terminated and not resumed.

**Fig. 1:**
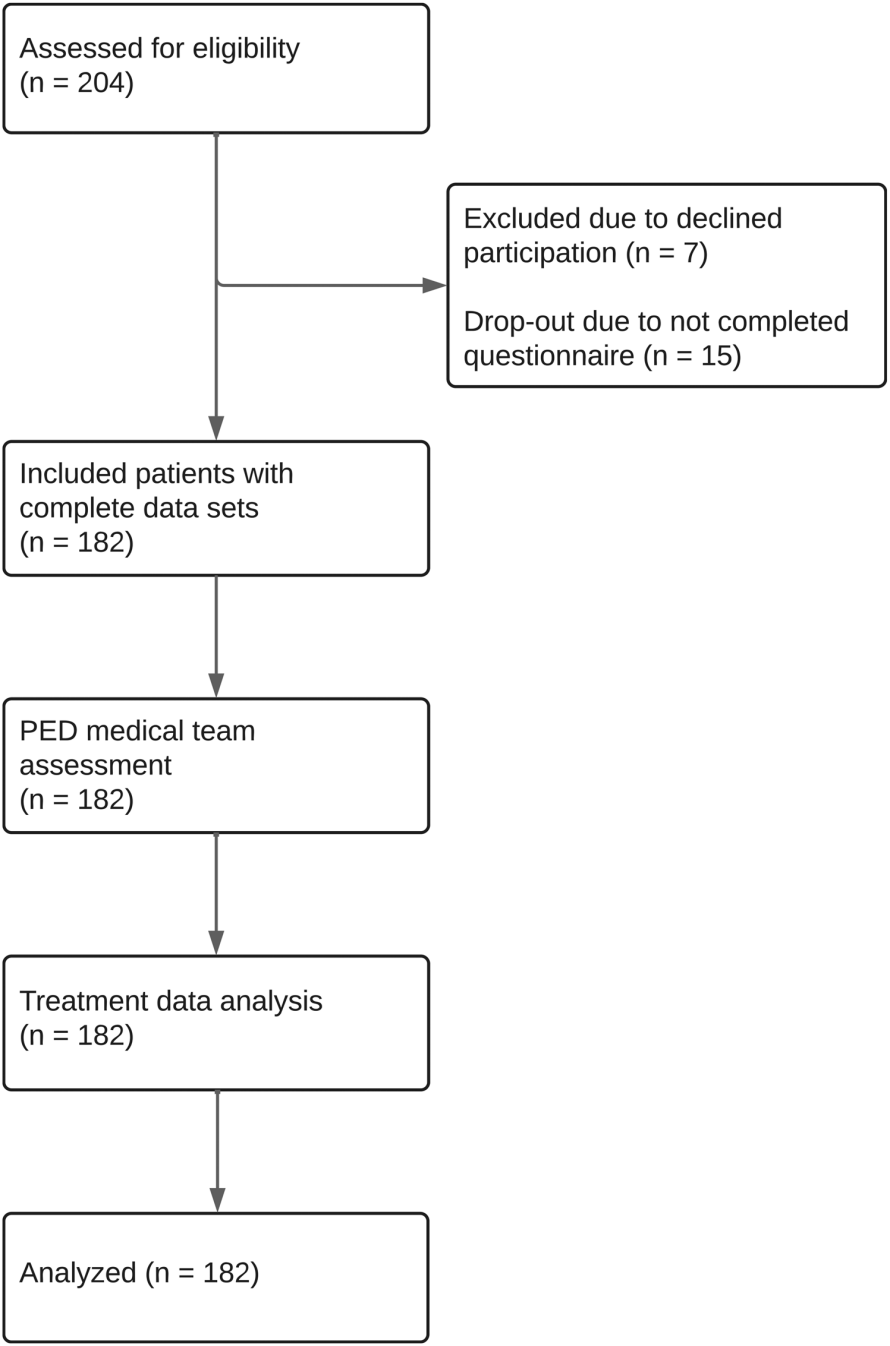
Consort-style diagram of data collection process (in concordance with STROBE [16]). After informed consent had been obtained, included patients filled in the questionnaire. After the patient’s initial interaction with the medical personnel (PED nurse and physician), both professionals independently assessed the severity of the patient’s emergency condition. Subsequently, the actual condition severity was assessed by analyzing treatment data

Following questionnaire completion, the attending PED nurse and physician independently rated the emergency severity based on their first impression. They were not permitted to discuss the patient prior to providing their assessments. The composition of the medical team varied between shifts. Cases were excluded from analysis if multiple interactions occurred between the patient and the medical team before the assessments to prevent mutual influence. Thirty days after admission, emergency medicine specialists conducted a retrospective review of medical records to obtain each patient’s final diagnosis and, where applicable, details of their treatment during hospitalization (i.e. retrospective case assessment. A flowchart illustrating the data collection process is provided in Fig. 1.

### Survey contents and outcome measures

#### Patient

The questionnaire for patients (Supplementary 2) was illustrated to compensate for their potential inability to read. Only patients older than 4 years of age completed the questionnaire, with assistance from the study team if necessary. The questionnaire contained three ordinal numeric scales ranging from 0 to 10 including facial depictions to determine the patients’ subjectively felt pain, sickness, and anxiety – based on the Numeric Rating Scale [17]:

*“Are you experiencing pain right now? If so*,* how severe would you rate this pain?”*; 0 (no pain) to 10 (unbearable extreme pain).

*“How sick do you feel right now?”*; 0 (not sick) to 10 (very sick).

*“Are you currently afraid because you are ill? If so*,* how intense would you say this fear is?”*; 0 (not afraid) to 10 (very afraid).

Additionally, the patients were asked for their assessment of six food items to evaluate their FL (“*Which pictures do you think show healthy food? Please tick the boxes.”*).

#### Parent

The questionnaires for the parents consisted of three parts: They were requested to provide a subjective assessment of the emergency condition severity of their child on an ordinal scale: “*How threatening do you perceive your child’s current emergency condition to be for your child’s health?*” The scale ranged from 1 (barely threatening) to 10 (life-threatening). Additional questions are presented in Supplementary 3. The second part consisted of the German version of the HLS-EU-Q16 (Supplementary 4), a well-established and validated tool to acquire an individual’s HL [18]. The results determine an individual’s HL level, which is classified into three categories: inadequate, problematic, or adequate HL. Inadequate and problematic HL can collectively be referred to as limited HL [18], a term often used interchangeably with low HL. HLS-EU-Q16 does not allow conclusions about specific decision-making abilities in medical emergencies; its focus is on general and preventive health-related knowledge. However, it is one of the most validated tools for measuring health literacy. The third section of the questionnaire gathered demographic information, including educational background (secondary school, high school, college, or university) and age. In Germany, college and university represent distinct educational pathways.

#### PED medical team

The PED medical team subjectively assessed the severity of the emergency upon presentation.

PED physicians (*n* = 8) had an average of three years’ experience (range: 2–6 years), while most PED nurses (*n* = 15) had over 12 years of experience (97%). First contact with the PED physician typically lasted around 10 min, while contact with PED nurses was usually under 10 min.

Immediately following their initial contact with the patient, the medical team (PED nurse and physician) independently provided a subjective assessment of the patient’s condition severity using an ordinal scale. They were asked: “*How seriously ill or injured is this patient right now?*” The scale ranged from 1 (barely threatening) to 10 (life-threatening). The duration of the team’s interaction with the patient (in minutes) and their professional experience (in years) were also documented.

The 10-point ordinal severity scale was adapted from validated numeric rating scales (e.g., NRS for pain). It shows moderate-to-strong correlations with objective outcomes in similar ED studies (e.g., ρ = 0.45 with admission, prior adult study ρ = 0.52) [15]. Initial pilot testing (*n* = 20) confirmed feasibility and alignment with ESI categories (ρ = 0.58, *p* < .01). The ESI triage assessments could not be used as they are based on resource requirements rather than perceived threat.

#### Case assessment by specialists and high severity cases

Each patient case was reviewed 30 days after presentation by four pediatric emergency medicine specialists (“Specialists”) using medical records from the clinical information system. The Specialists had an average of 12.1 years of professional experience (range: 10–15 years). Only data spanning from the day of PED presentation to 30 days post-presentation was included in the analysis. All Specialists worked independently and were blinded to potentially confounding contextual factors (e.g., presentation day or triage category). The interrater agreement among the Specialists was 93%.

Their retrospective assessments were recorded on an ordinal scale ranging from 1 (barely threatening) to 10 (life-threatening), representing the perceived severity of each emergency. This assessment is based on the extensive experience of the specialists but remains a subjective assessment. Examples of severity assessments are provided in Supplementary 5.

High severity was defined in two ways: based on the distribution and based on key points of the clinical course. We defined a high severity (i.e. Severe Outcome) distribution-based corresponding to specialist assessments at least one standard deviation above the cohort mean (M = 4.45, SD = 1.25; cutoff = 5.70). This approach corresponds to typical triage systems, such as the Emergency Severity Index used in our PED, which allocate the highest two categories to approximately the top 10–20% of cases, reflecting an M+1SD cut-off [19, 20]. The results aligned with expectations, with high severity (≥ 5.70, 6 on ordinal scale, i.e., Mean + 1 SD) observed in 15% (*n* = 28) of cases. We additionally defined high severity based on key point3s of the clinical course. Cases with distribution-based Severe Outcome always met at least two of the following specific clinical markers in any case: monitoring of vital signs for more than 12 h, inpatient admission for more than 24 h, care escalation within 24 h, ICU admission, application of parenteral antibiotics, surgical procedure or invasive intervention within 72 h. See Supplementary 6.

Accordingly, scores ≥ 6 on the ordinal scale were considered indicative of conditions posing a persistent danger to the patient’s health and thus classified as Severe Outcome. This classification was robust regarding our outcome measures, as alternative thresholds (i.e., M+1SD, top quintile, only clinical markers) did not change the results. Retrospective specialist assessments (blinded to ESI) showed strong convergence, with scores ≥ 6 aligning with ESI levels 1–2 in 85% of cases.

#### Discrepancy

Discrepancy represents the difference between the patient’s or parent’s assessment and the evaluations provided by the PED medical team or specialists. A greater deviation - positive or negative - from 0 indicates a larger divergence, reflecting reduced predictive accuracy of the patient’s self-assessment. Negative values below − 1 denote an underestimation of condition severity by the patient, referred to as a ‘Negative Discrepancy’. Conversely, positive values above 1 indicate an overestimation compared with professional assessments, termed a ‘Positive Discrepancy’. Values between − 1 and 1 were considered to demonstrate ‘Concordance’ between the patient’s and professionals’ assessments. Concordance represents a pragmatic definition of clinically acceptable deviation. To test robustness, we repeated all discrepancy analyses using stricter (-0.5 to 0.5) and more lenient (-2 to 2) Concordance thresholds including agreement indices (see Supplementary 7). For statistical analysis, absolute values were used to quantify the degree of (dis)agreement, while actual values were applied to determine its direction.

Four variants were used: Discrepancy (Patient/Medical Team), Discrepancy (Patient/Specialists), Discrepancy (Parent/Medical Team), and Discrepancy (Parent/Specialists). For the medical team assessments we used the mean of the nurse’s and physician’s severity assessments to approximate a team-level clinical consensus. This aggregation is consistent with our prior adult ED study and reflects routine interdisciplinary decision-making in the PED. To calculate Discrepancy, the average of the assessments of the PED physicians, the PED nurses or the specialists was subtracted from the patient’s or parent’s assessments:

**Table:** 

**Discrepancy** (**Patient-Medical Team)** =	Patient – (*Mean* (PED Physician, ED Nurse))
**Discrepancy (Patient-Specialists)** =	Patient – (*Mean* (Specialists))
**Discrepancy (Parent-Medical Team)** =	Parent – (*Mean* (PED Physician, ED Nurse))
**Discrepancy (Parent-Specialists)** =	Parent – (*Mean* (Specialists))

#### Food literacy

HL in children and adolescents cannot be directly assessed; it must be inferred through their parents. We considered FL may serve as a practical proxy variable for children’s HL, because it is easily available even at an early age. The link between FL and HL in children is scarcely investigated, although FL is considered part of HL. In this study, patients have been asked for their assessment of six illustrated food items. The pictures tested their food-related knowledge and was designed for a broad age range (4–17 years). See Supplementary 2.

#### Data analysis

The minimum required sample size was determined to be 180 patients (see Supplementary 8). All data were analyzed using descriptive statistical methods. Normality was assessed using the Shapiro–Wilk test and Q–Q plots. Spearman’s rank-order correlations (ρ) were calculated to quantify concordance between raters, with Fisher’s z values indicating effect sizes. Discrepancies were computed as both signed and absolute differences and compared across parental HL levels using Kruskal–Wallis tests. Significant omnibus effects were followed by Dunn post-hoc tests with Holm correction, and η² was reported as the effect size. Severe outcomes were defined as mean specialist ratings ≥ 1 SD above the overall sample mean. HL was analyzed both categorically (inadequate, problematic, adequate) and continuously (sum score 0–16), with 95% CIs for group differences. Binary logistic regression models were used to predict severe outcomes based on HL, rater assessments, and relevant covariates, reporting odds ratios (OR), 95% confidence intervals (CI), and R² values. All statistical analyses were conducted using SPSS Statistics version 29.0.0 (build 241; IBM, New York, USA) in collaboration with a faculty biostatistician who remained blinded to study conditions. Figures were generated using Prism version 10.2.1 (build 339; GraphPad Software, LCC, Boston, USA). Unless otherwise specified, all results were evaluated using a two-sided significance level of α = 0.05. Details of the biometrical approach are provided in Supplementary 9.

Condition severity assessments were adjusted for covariates (Age, Education, and Gender for parents; Age for patients), as described in Supplementary 10. Regression analyses were performed using unadjusted values.

### Data Availability

The data generated and analysed during the current study are available from the corresponding author on reasonable request.

## Results

This study examines whether low HL affects a parent’s ability to accurately assess the severity of their child’s emergency condition and whether low HL contributes to greater discrepancies between parental or patient assessments and those of medical professionals. Furthermore, it explores whether higher HL is associated with lower levels of such discrepancy.

### Characteristics of participants

204 patients were eligible for inclusion. 197 patients and parents agreed to participate. 15 were later excluded due to incomplete questionnaires. This was due to organizational procedures in the PED (e.g. call-up for X-ray examination) in all cases. 182 patients therefore had a complete data set (94%). Only the latter were used for analysis, see Table 1. In the following explanations, the term ‘parent’ always includes legal guardians.

The mean age of included patients was 8.67 years (Md 9, SD 4.95, range: 0–17 years). More patients were male (60%) and most often accompanied by their mother (73%). The mean age of their parents was 39 years (SD 7.61, range: 19–72). Most parents had completed university (42%). This distribution is common for the local PED yet it is roughly 5% higher than German average [21]. The overall distribution of diagnoses in the patients included was representative of the common reasons for presentation to our PED, see Supplementary 6.

Patients and parents were categorized into three HL groups based on parents’ HLS-EU-Q16 questionnaire results: adequate (*n* = 85, 47%), problematic (*n* = 76, 42%) and inadequate (*n* = 21, 11%). Limited HL (combining problematic and inadequate HL [18]) was prevalent in 53% of cases and therefore below the German average (59%) [22]. There were no demographic differences between the groups. See Table 1 for a detailed group description.

**Table 1:** HL group characteristics; values presented as count (largest share as percentage). Non-adjusted emergency condition severity assessments by Patient, Parent, PED Nurse, and PED Physician, categorized by HL group; median (SD). Chart-based retrospective case assessment by emergency medicine specialists; median (SD). HLS-EU-Q16 Sum-score; mean (SD). Presentation time and day; count (largest share as percentage)

	All (*n* = 182)	HL inadequate (*n* = 21, 11%)	HL problematic (*n* = 76, 42%)	HL adequate (*n* = 85, 47%)
**Patients**				
Female	73	11 (52%)	32	30
Male	109 (60%)	10	44 (57%)	55 (65%)
*Age*,* in years*				
Mean	9 (4.95)	8 (4.63)	9 (4.95)	9 (4.97)
Range (in years)	0–17	1–17	0–17	0–17
*Age groups*,* count*				
0–3 years	39	5 (24%)	17	17
4–6 years	27	4	10	13
7–9 years	33	4	13	16
10–13 years	47 (26%)	4	21 (27%)	22 (26%)
14–17 years	36	4	15	17
**Parent**				
Female	132 (73%)	18 (86%)	55 (72%)	60 (70%)
Male	49	3	21	25
Age, in years	39 (8.16)	39 (5.77)	41 (7.72)	39 (8.05)
Age range, in years	19–72	26–47	23–72	19–62
Parent BMI	26 (5.28)	30 (6.15)	25 (4.62)	26 (4.84)
*Education*:				
Secondary general school	24	2	9	13
Secondary school	56	10 (48%)	21	25
Academic secondary school	25	1	9	15
University	77 (42%)	8	37 (49%)	32 (38%)
**Condition severity assessment**				
Patient	3 (2.65)	4 (2.74)	4 (2.73)	3 (2.49)
Patient – pain assessment	5 (2.56)	4 (2.38)	3 (2.50)	3 (2.60)
Patient – anxiety assessment	2 (2.63)	1 (2.79)	2 (2.73)	2 (2.53)
Parent	5 (1.98)	6 (2.48)	5 (1.88)	4 (2.02)
PED nurse	4 (1.54)	4 (1.66)	4 (1.58)	4 (1.51)
PED physician	3 (1.34)	3 (1.72)	3 (1.25)	3 (1.32)
**HLS-EU-Q16 Sum-score**	11.89 (2.66)	6.85 (1.46)	10.66 (1.06)	14.19 (1.01)
**Chart-based case assessment**				
Specialists	4 (1.43)	4.17 (1.64)	4.00 (1.51)	4.00 (1.28)
**Time to the ED** (count)				
10–12 AM	26	2	11	13
12 AM-2 PM	26	3	11	12
2–4 PM	37	3	16	18
4–6 PM	42	6	17	19
6–8 PM	51 (28%)	7 (33%)	21 (28%)	23 (27%)
**Weekday to the ED**				
Monday	18	3	7	8
Tuesday	19	1	8	10
Wednesday	22	2	9	11
Thursday	16	1	6	9
Friday	27	3	10	14
Saturday	42 (23%)	5	19 (25%)	18 (22%)
Sunday	38	6 (29%)	17	15

Abbreviations: HL, Health literacy; PED, Paediatric Emergency Department

### Parental health literacy and accuracy of severity assessments

We examined whether parental HL influences the accuracy of parental and child severity assessments using group comparison, correlational, and regression analyses across the entire sample and grouped by HL levels.In the complete sample, parental HL demonstrated no measurable associations with condition severity assessments. HL (i.e., HLS-EU-Q16 score) did not correlate significantly with any measure of discrepancy, regardless of whether discrepancy was defined as disagreement between parents and the medical team, parents and specialists, or children and professionals (all |ρ| < 0.12, all *p* > .10). Continuous HL sum showed no relevant associations with absolute discrepancies (all |ρ| < 0.07, all 95% CIs include 0, all *p* > .12). Categorical group differences yielded non-significant results (all *p* > .16), with 95% CIs for inadequate vs. adequate HL overlapping zero (e.g., Parent–Specialists Δ = 0.37 [− 0.29, 1.02]). Multivariate regression models predicting discrepancy from parental HL, parent age, education, and gender confirmed these findings: across all models, HL was not a significant predictor (all *p* > .50, R² < 0.02). Parents across all HL levels demonstrated similar estimation patterns relative to professional assessments.

Group comparisons across the three HL levels revealed no significant differences in Parent–Medical team discrepancy (*H*(2) = 3.81, *p* = .149) or Parent–Specialists discrepancy (*H*(2) = 3.02, *p* = .221), see Table 2. The distribution of discrepancy directions was similar across HL groups, with positive discrepancies predominating in all three categories, see Table 3. Parents across all HL categories more frequently overestimated their child’s illness severity relative to professional judgments. In the adequate HL group, 56% of parents rated illness severity higher than the medical team. This proportion decreased to 51% in the problematic HL group and increased to 72% in the inadequate HL group. The groups did not differ significantly in Patient–Medical team or Patient–Specialists discrepancies (*p* > .05). It must be noted that inadequate HL (*n* = 20) was considerably less frequent than problematic or adequate HL as was to be expected. Patients tended to underestimate condition severity across parental HL-levels.

When examining the clinical significance of these discrepancies, Severe Outcome rates showed no consistent HL-related gradient, see Table 3. Severe Outcome cases occurred at comparable rates across HL levels and discrepancy categories, with no systematic advantage or disadvantage for higher HL. Thus, low HL neither impaired the recognition of condition severity nor systematically increased disagreement with clinicians. Concordance rates were similar across HL levels.

Sensitivity analyses using alternative concordance thresholds (stricter ± 0.5, more lenient ± 2) confirmed the absence of systematic HL effects and the association between underestimation and Severe Outcome (Supplementary 7). In summary, parental HL showed no measurable association with the accuracy or direction of parental or child severity assessments. Across all HL levels, parents displayed similar patterns of overestimating their child’s illness severity relative to professionals; Severe Outcome rates did not differ systematically by HL or discrepancy category, and low HL neither impaired recognition of severity nor increased disagreement with clinicians.

**Table 2:** Group (HL level) comparison regarding Discrepancy. Discrepancy between parent’s or patient’s and PED medical team’s or emergency medicine specialists’ assessments, mean (SD). *Asterisk marks significant comparisons

Discrepancy	Overall	Inadequate HL	Problematic HL	Adequate HL	
**Parent**					
Medical Team	1.31 (1.97)	2.01 (2.00)	1.12 (1.92)	1.32 (1.99)	H(2) = 3.81 *p* = .149
Medical Team (absolute)	1.90 (1.40)	2.42 (1.46)	1.76 (1.35)	1.91 (1.43)	H(2) = 3.55 *p* = .169
Specialists	0.54 (2.24)	1.24 (2.19)	0.33 (2.41)	0.55 (2.08)	H(2) = 3.02 *p* = .221
Specialists (absolute)	1.87 (1.35)	2.08 (1.37)	1.98 (1.40)	1.71 (1.30)	H(2) = 2.08 *p* = .353
**Patient**					
Medical Team	−0.13 (2.82)	1.06 (2.78)	0.11 (3.10)	−0.61 (2.49)	H(2) = 4.72 *p* = .094
Medical Team (absolute)	2.29 (1.63)	2.56 (1.33)	2.51 (1.79)	2.04 (1.54)	H(2) = 2.35 *p* = .308
Specialists	−0.90 (2.98)	−0.01 (2.70)	−0.73 (3.17)	−1.28 (2.81)	H(2) = 3.23 *p* = .199
Specialists (absolute)	2.54 (1.78)	1.95 (1.78)	2.61 (1.90)	2.58 (1.68)	H(2) = 1.74 *p* = .418

Abbreviations: HL, Health literacy; PED, Pediatric emergency department

**Table 3:** Group characteristics regarding discrepancy. Discrepancy describes the deviation between the parent’s or patient’s assessment and the assessment of the PED medical team or emergency medicine specialists in two different combinations: Discrepancy Medical Team and Discrepancy Specialists. Bolded values indicate the most frequent feature within this group; presented as percentage of group. = Equal indicates Concordance, + Plus indicates Positive Discrepancy, and - Minus indicates Negative Discrepancy. Severe Outcome of total describes the proportion of severe cases (Specialists’ assessment > 5.70) as percentage on this HL level. Each cell below shows the number of Severe Outcome within that discrepancy category, the HL level, and the direction of discrepancy

Discrepancy	Inadequate HL (*n* = 20)			Problematic HL (*n* = 77)			Adequate HL (*n* = 85)		
	=	+	−	=	+	−	=	+	−
**Parent**									
Medical Team	17%	**72%**	11%	32%	**51%**	17%	30%	**56%**	14%
Specialists	32%	**53%**	16%	30%	**37%**	33%	38%	**39%**	22%
**Patient**									
Medical Team	18%	**64%**	18%	21%	36%	**43%**	31%	25%	**44%**
Specialists	25%	**50%**	25%	26%	30%	**44%**	18%	24%	**58%**
***Severe Outcome***	of total: 3 (15%)			of total: 14 (18%)			of total: 11 (13%)		
**Parent**									
Medical Team	1	2	0	3	8	3	4	5	1
Specialists	1	1	1	4	1	9	6	0	5
**Patient**									
Medical Team	0	2	1	2	5	5	2	1	4
Specialists	1	1	1	3	1	8	1	0	7

Abbreviations: HL, Health literacy; PED, Pediatric emergency department

### Condition severity assessments

Across the entire sample, patients’ mean self-assessment of emergency condition severity was 3.5 (SD = 2.62, Md = 3, range: 0–10). Parents’ mean assessment of their child’s condition was 4.2 (SD = 2.02, Md = 5, range: 1–9), indicating that parents rated the condition approximately 20% more severe than their children. The pediatric nurses’ mean assessment was 3.9 (SD = 1.51, Md = 4, range: 1–9), while pediatric physicians’ mean assessment was 3.4 (SD = 1.35, Md = 3, range: 1–8). Results stratified by HL levels are presented in Table 1. No significant differences in any assessment measure emerged between parents’ HL levels (*p* > .05).

#### Case assessment

The following data are based on retrospective case assessments conducted by pediatric emergency medicine specialists using chart review. Inter-rater agreement between emergency specialists was 93%. The mean severity rating for all included patients was 4.45 (SD = 1.25, Md = 4, range: 1.67–10). No significant differences were observed across HL levels or age groups (*p* > .05). Correlation analysis revealed positive associations between specialists’ case assessment and both pediatric physicians’ assessments (ρ = 0.20, *p* < .01, 95% CI [0.04, 0.34], z = 0.20) and pediatric nurses’ assessments (ρ = 0.26, *p* < .001, 95% CI [0.11, 0.42], z = 0.26). In contrast, patients’ and parents’ severity assessments did not correlate with specialists’ assessments (*p* > .05). (Quadratically) weighted κ corroborated the Spearman correlations (Supplementary 11). Overall, the case assessment analysis yielded results consistent with expectations, with Severe Outcome (≥ 6) occurring in 15% of cases (*n* = 28).

#### Patient’s pain and anxiety

The mean patient pain rating across the entire sample was 4.4 (median = 5.0, SD = 3.06, range: 0–10). The mean anxiety rating was 2.96 (median = 3.0, SD = 2.71, range: 0–10). No significant differences emerged between age groups or parental HL levels (*p* > .05).

In summary, parents across the whole sample rated their child’s condition severity about 20% higher than patients themselves, while specialists’ assessments correlated with pediatric physicians’ and nurses’ evaluations but not with parent or patient assessments. No significant differences emerged by parental HL or age group in severity, pain, or anxiety ratings.

### Age-dependent validity of patients’ self-reports

**Fig. 2:**
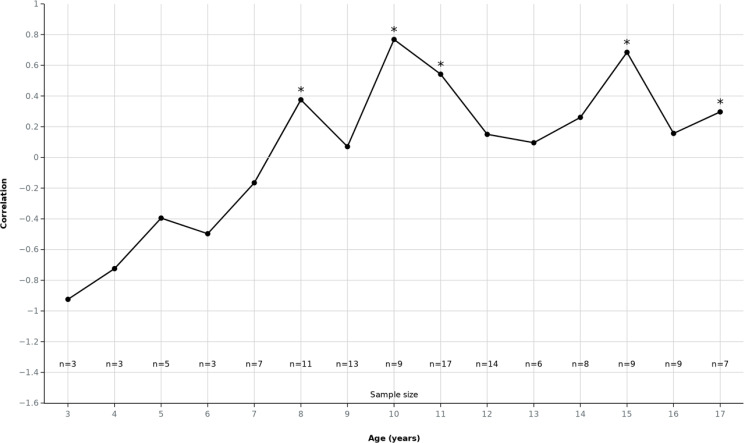
Depiction of developmental trend in the validity of children’s anxiety self-assessments as indicators of clinical severity. Spearman correlation coefficients between patient-reported anxiety and specialist-evaluated condition severity across patient ages 3 to 17 years. Data points represent the strength and direction of correlations by age, with asterisks (*) indicating statistically significant correlations (*p* < .05). The sample size (n) is stated above each age

We investigated how developmental age influences the clinical validity of children’s self-reported emergency condition severity, pain, and anxiety. The analysis aimed to identify the developmental threshold at which children’s self-assessments begin to align reliably with professional evaluations. Among children below age 6 years (i.e., preschool-age), concordance between patient self-assessment and the medical team was low (ρ = 0.04) but improved in the 7–9 age group (ρ = 0.30). This developmental pattern suggests a gradual improvement from early school age onward (around 7–8 years), rather than a sharp threshold at a single age, with correlations and interaction effects reaching statistical significance first in this age range. Children’s conceptualization of illness may become sufficiently developed to permit valid self-assessment around this age. A logistic regression model predicting Severe Outcome using patient condition severity assessment, pain, and anxiety ratings, as well as age, was significant (χ²(4) = 28.6, *p* < .001, R² = 0.28): patient condition severity assessment (*p* < .001; OR = 1.38, 95% CI [1.20, 1.57]) and anxiety (*p* = .037; OR = 1.15, 95% CI [1.01, 1.31]) emerged as significant predictors, while pain and age did not. When interaction terms were added, a significant Age × Anxiety interaction was detected (*p* = .022): beginning around age 7, anxiety becomes a reliable and increasingly potent indicator of clinical severity. No comparable age moderation was observed for pain or self-rated illness severity (both *p* > .40), see Fig. 2.

In summary, children’s self-reports of condition severity, pain, and anxiety show low concordance with professional assessments before age 6 but improve notably after age 7, with anxiety emerging as a significant and increasingly reliable predictor of clinical severity starting around this developmental threshold.

### Predictive power regarding severe outcome

We examined predictors of Severe Outcome, operationalized as unadjusted specialists’ ratings at or exceeding 5.70. Logistic regression models included patient-reported condition severity, pain, anxiety, and age as predictors. Only unadjusted values were used. The model was significant (χ²(4) = 28.6, *p* < .001), explaining approximately 28% of the variance in severe outcomes. Patient-reported condition severity (OR = 1.38, 95% CI [1.20, 1.57], *p* < .001) and anxiety (OR = 1.15, 95% CI [1.01, 1.31], *p* = .037) were significant predictors, whereas pain (*p* = .34) and age (*p* = .23) were not. To assess potential age moderation, a second model was estimated that included interaction terms for Age × Patient (condition severity), Age × Pain, and Age × Anxiety. The Age × Anxiety interaction was significant (B = 0.071, SE = 0.031, *p* = .022), indicating that anxiety increasingly predicts severe outcomes with advancing developmental age. The remaining interaction terms were nonsignificant (*p* > .40). Predicted probabilities derived from this model illustrate the developmental progression: at age 5, anxiety exerted minimal influence on predicted severity (probability approximately 0.15–0.25). At age 7, this relationship strengthened noticeably, with predicted severity probabilities ranging from 0.18 at low anxiety to 0.45 at high anxiety. By age 10, the same anxiety increment corresponded to severity probabilities ranging from 0.20 to 0.70. Thus, beginning around age 7, children’s anxiety reports increasingly reflect actual clinical severity in a consistent, dose-dependent manner. In younger children, anxiety may primarily reflect global distress or contextual fear unrelated to illness severity, whereas in older children, anxiety becomes increasingly linked to specific perceptions of illness severity.

**Fig. 3:**
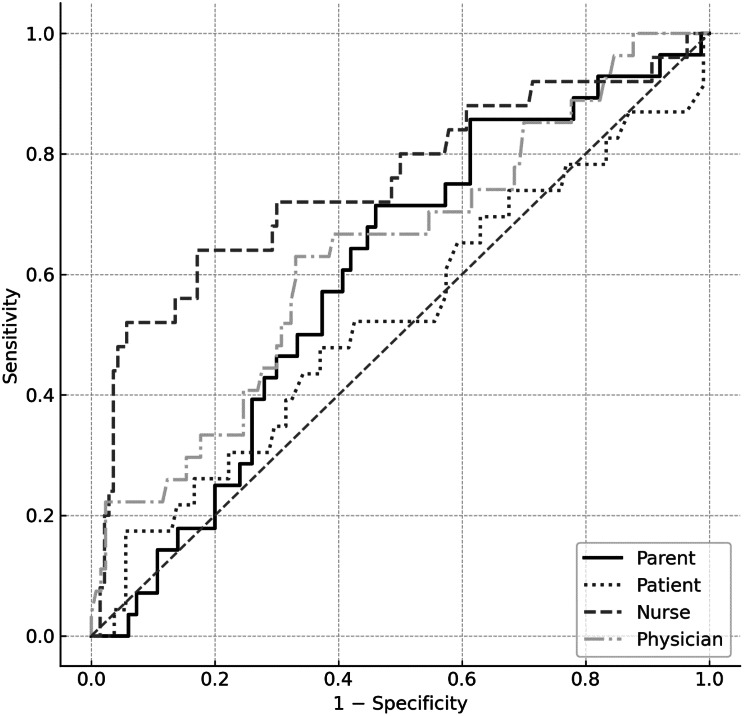
ROC curves for condition severity ratings by parents, patients, PED nurses, and PED physicians predicting severe outcome (specialist rating ≥ M + 1 SD). The diagonal line represents chance-level discrimination. Nurses’ ratings show the highest discriminative accuracy (AUC ≈ 0.75), followed by physicians (AUC ≈ 0.64). Parents’ (AUC ≈ 0.58) and patients’ (AUC ≈ 0.53) accuracy was lower

Among professional raters, pediatric nurses demonstrated consistently high predictive validity. A combined logistic regression model including parents’, patients’, nurses’, and physicians’ condition severity assessments as predictors of Severe Outcome was highly significant, explaining approximately half the variance (R² = 0.48). Nurses’ ratings emerged as the strongest unique predictor (OR = 1.95, 95% CI [1.37, 2.67], *p* < .001), followed by physicians (OR = 1.62, 95% CI [1.20, 2.19], *p* < .001). Patients assessments were no strong yet a significant predictor (OR = 1.25, 95% CI [1.02, 1.48], *p* = .007). Once other evaluations were included in the model, parents’ ratings did not significantly predict severity (*p* = .216). Receiver operating curves (ROC) analysis confirmed the differences in the predictive accuracy of the rater groups. Nurses’ assessment demonstrated the highest discriminative ability (area under the curve, AUC ≈ 0.75, *p* < .001), reliably distinguishing severe from non-severe cases. Physicians’ ratings showed moderate predictive performance (AUC ≈ 0.64, *p* < .001), whereas parents’ and patients’ ratings exhibited only limited accuracy (AUC ≈ 0.58/53, *p* < .05), see Fig. 3.

Regarding the predictive power of discrepancy, we used signed discrepancy values to classify direction and observed complementary patterns across the entire sample. Discrepancy direction and extent varied only slightly by HL level but underestimation by parents or patients consistently correlated with higher rates of severe outcomes, see Table 3. Positive discrepancy (overestimation) was more common among parents across HL groups, though severe outcomes were lower in this category. Underestimation (negative discrepancy) showed elevated severe outcomes, particularly in parent–specialist comparisons. Concordance rates were highest in the adequate HL group but did not protect against underestimation risk. These findings highlight the critical impact of underestimation on severe outcomes regardless of HL level. However, HL did not systematically alter these patterns. When data were stratified by HL level, the distributions for all discrepancy categories showed no significant differences (both *p* ≥ .05). Severe Outcome varied across HL groups without demonstrating a coherent gradient. Negative Discrepancies most frequently associated with Severe Outcome, whereas Positive Discrepancies corresponded to the lowest rates of clinical severity.

In summary, both parental and patient underestimation of condition severity relative to specialists served as meaningful indicators of truly severe cases, while overestimation generally reflected less critical conditions. Importantly, HL did not systematically influence these relationships, suggesting that the clinical significance of discrepancy direction is comparable across HL levels.

### Patient’s food literacy

A total of 141 patients completed the FL questionnaire. The maximum achievable score was 8. Most patients scored above 7 points (*n* = 104; M = 7.2, SD = 0.41), showing a dominant ceiling effect with nearly no variance. No significant correlation was found between parents’ HL scores and patients’ FL scores (*p* > .05). In this form, the acquisition of FL is not informative, most likely due to an overly simple test.

## Discussion

This study examined how parental HL influences the accuracy of severity assessments in pediatric emergency care and identified child age as a critical determinant of self-report validity. Using data from parents, pediatric patients, PED nurses, PED physicians, and pediatric emergency medical care specialists, we investigated which perspectives best predict clinically severe outcomes. Contrary to our expectations [14], parental HL showed no detectable effect on assessment accuracy or agreement with clinicians within the limitations of this study. Child age emerged as the key developmental factor determining validity of self-reported symptoms. Finally, the professional assessment of nurses remained the most reliable indicator of critical illness.

Parental HL demonstrated no significant association with discrepancies from professional assessments. Parents with lower HL did not systematically underestimate or overestimate their child’s condition, and their ability to recognize severe cases matched that of parents with higher HL. Yet in adults’ self-assessment, low HL can impair judgment in emergency situations [15]. These findings suggest that in emergency contexts concerning the offspring, literacy-related differences may be attenuated by other factors such as emotion or uncertainty [23, 24]. Similar to results of other study [25, 26], our findings show that parents tended to overestimate their child’s severity in comparison to clinicians. This might be driven by psychological factors, rather than medical knowledge and can also be influenced by a lack of experience leading to misinterpretations of symptoms [27]. Factors such as situational stress, prior experiences, or contextual cues might also influence judgment.

For this study, several mechanisms are likely to explain this unexpected result. (Pediatric) Emergency departments provide rich contextual and behavioral cues like medical staff reactions that help parents interpret their child’s condition independent of formal health knowledge. Parental assessments are often grounded in close familiarity with their child’s usual behavior [28], offering intuitive understanding that may compensate for limited HL. Additionally, clinicians frequently adapt communication to families’ comprehension levels, mitigating disadvantages associated with lower HL. This is particularly true in the specific area of pediatric emergency care, as even well-informed parents can quickly become overwhelmed in such situations. These factors suggest that while HL is relevant, its influence on acute severity perception during pediatric emergency visits is limited.

Importantly, parental underestimation compared to (retrospective) specialist evaluations predicted genuine clinical severity. Severe outcomes occurred in 33% of underestimation cases but only in 3% with overestimation. The frequency and direction of these discrepancies did not vary significantly across HL levels, reinforcing that literacy does not fundamentally alter parental perceptual alignment with clinicians. An explanation for this observation could be that underestimation on the part of parents might be connected to emotional denial in acute situations.

Patient (i.e., child) age proved decisive in determining the reliability and predictive power of self-reported symptoms. Correlations between children’s and clinicians’ severity ratings followed a clear developmental path: inconsistent in preschoolers, moderate by middle childhood, and increasingly variable thereafter. Both self-rated severity and anxiety independently predicted severe outcomes, but anxiety’s predictive effect increased significantly with age. The significant age and anxiety interaction emerged at approximately 7–8 years, marking a developmental threshold where anxiety became a meaningful clinical signal. Earlier studies also identified school-age as threshold for valid self-report [13]. School-age children can provide increasingly reliable self-assessments of pain and health status [29, 30], coinciding with the transition to concrete operational thinking and improved capacity to link internal states to illness concepts [31]. This developmental threshold corresponds to established milestones in cognitive maturation and school entry in Germany, when children typically gain enhanced self-conceptualization and verbal expression of internal states. While exact statistical significance varied by single age year (e.g., strongest correlations emerging at age 8), a clear and consistent trend toward improved validity of self-reported anxiety and severity assessment was evident from early school age onward. This pattern supports age-appropriate integration of pediatric patient input in clinical risk stratification.

This developmental change likely reflects advances in the understanding of body signals. Before age seven, anxiety can express nonspecific distress like separation anxiety rather than illness awareness. As children mature, they might increasingly differentiate symptom-related from situational anxiety and link emotional states to physiological sensations [32]. Anxiety might become a proxy for medical condition severity awareness after this cognitive differentiation develops. This fits in well with the Piaget’s Theory and Stages of Cognitive Development, more specifically the “Concrete Operational Stage”, in which children start to show logical thinking [33]. Pain ratings remained poor predictors across all ages, possibly because pain perception of children is obscured by contextual factors in emergency settings.

When modeling all four perspectives simultaneously, professional assessments clearly outperformed untrained evaluations. Nurses’ ratings were the strongest predictor of severe outcomes (OR = 1.95), followed by physicians (OR = 1.62) and patients (OR = 1.25). Parents’ ratings lost significance when others were included. Nursing assessments reached high precision possibly due to experience and continuous bedside observation as well as longer interactions with the patient [34–36]. Children’s self-assessment retained a small but significant effect even after controlling for professional evaluations, suggesting children provide meaningful information.

Our findings suggest that interventions to improve parental decision-making in emergencies may not require targeting HL directly but should instead enhance situational and emotional understanding. Practical examples include low-threshold waiting room materials such as icon-based traffic light systems guiding ‘When to seek emergency care?‘, mobile (web)apps or checklists incorporating symptom duration, child behavior, and anxiety cues, kindergarten/school programs teaching children age-appropriate pain and fear expression, and standardized PED communication strategies like safety explanations with ‘teach-back’ confirmation at discharge. These approaches prioritize decision support and might aid emotion regulation while leveraging intuitive parental strengths observed in this study.

Pediatric patients’ anxiety and perceived severity appear most reliable from age 7 onward. Institutional protocols can rely on PED nurses’ assessments as a predictive source. The accuracy of clinical severity perception in pediatric emergencies depends less on health knowledge than on development, proximity, and professional experience.

### Limitations

This study has several limitations. The patients were spread across a wide age range and, accordingly, a wide developmental range. This is not comparable to age differences among adult patients. The children’s development inevitably and consciously influences the results of the study. Further studies should examine specific age groups to determine causal relationships. The FL questionnaire in our study was designed from scratch. As nearly 75% showed a high score, we conclude that the pictural food questions were too easy. The questionnaire is therefore not a suitable tool in this form and needs to be adapted. HLS-EU-Q16’s ceiling effect and self-report (vs. demonstrated skills) nature limit the granularity, particularly in samples with higher results; the small inadequate HL group (*n* = 20) in our study reduced power for subtle effects. However, continuous collinearity checks confirmed robustness of null findings, but the results might favor detection of developmental patterns over subtle HL effects. Collinearity with education/age was minimal (Supplementary 12). Clinical outcomes were reported incompletely, as they constituted a secondary component of the emergency medicine specialists’ retrospective case reviews. Consequently, we did not evaluate delays in diagnosis, diagnostic errors, or adverse events. Although these endpoints are clinically important, they fall outside our primary research question. Our analysis focused on comparing subjective condition severity assessments across age and HL levels including their role for severe outcomes. Our data therefore cannot directly establish the causal effect of HL on emergency care outcomes. All subjective evaluations are inherently prone to bias. In our study, professional assessments were conducted by a dedicated expert team, which minimized biases such as confirmation bias and anchoring bias, although these biases cannot be eliminated. During retrospective case assessment, specialists operated independently and were blinded to contextual variables including arrival time and triage category. To ensure feasibility and comprehensive coverage, we enrolled only patients presenting from 10 AM to 8 PM. This sampling strategy may have introduced inclusion bias, as emergencies more common at night such as febrile convulsions were underrepresented. Other aspects, such as reduced resources or increased stress levels at night, may also have had an influence. Nevertheless, we consider the sample largely representative: it encompasses a large time period and captures the full spectrum of clinically relevant conditions. Additionally, the demographic characteristics of our participants including gender, age, HL level, education, and timing of PED visit are consistent with earlier epidemiological surveys. Due to the non-inclusion of critically ill patients requiring immediate intervention and those who arrived by EMS, particularly severe cases are underrepresented overall. However, in order to maintain the feasibility of the study during routine operations, no other type of inclusion was possible.

Ideally, subjective assessments of condition severity would have been obtained prior to triage to minimize procedural bias and unconscious influence from clinical staff. This was not feasible within the constraints of routine emergency department operations. However, the triage process in our PED does not routinely include distinct feedback and the ESI category is not communicated by default.

### Conclusion

Parental HL did not affect accuracy or discrepancies regarding condition severity evaluations. Underestimation signaled higher risk, as can be expected. Children’s self-assessment of illness and, from around early school age on, anxiety predicted severe outcomes properly while pain did not. In multivariable models, nurses outperformed physicians, patients, and parents in predicting severe outcome. Children’s FL did not correlate with parental HL or severity. Age-appropriate use of childrens’ self-assessments could further support risk stratification in the PED.

## Supplementary Information

Below is the link to the electronic supplementary material.

Supplementary Material 1

## Data Availability

The data generated and analysed during the current study are available from the corresponding author on reasonable request.

## Abbreviations

ED: Emergency Department
FL: Food Literacy
HL: Health Literacy
PED: Pediatric Emergency Department
