# Supplementary material for: Greater parental health literacy does not improve assessment of their child’s emergency condition severity
Source: BMC Emerg Med. 2026 Mar 17;26:99. doi: 10.1186/s12873-026-01541-8 (PMC13064169; doi:10.1186/s12873-026-01541-8)
Supplement: Supplementary file 1 — Supplementary Material 1 [file 12873_2026_1541_MOESM1_ESM.docx]

# Supplementary

## Randomization of sampling time slots:

Patient cases were included daily during study hours, which alternated randomly between five time slots: 10-12 AM, 12 AM-2 PM, 2-4 PM, 4-6 PM, 6-8 PM. The randomization for these time slots was implemented using permuted block randomization with dice rolls resulting in a predefined schedule and equally distributed time slots. Measurements were only taken during one time slot each day.

## Patient questionnaire

| 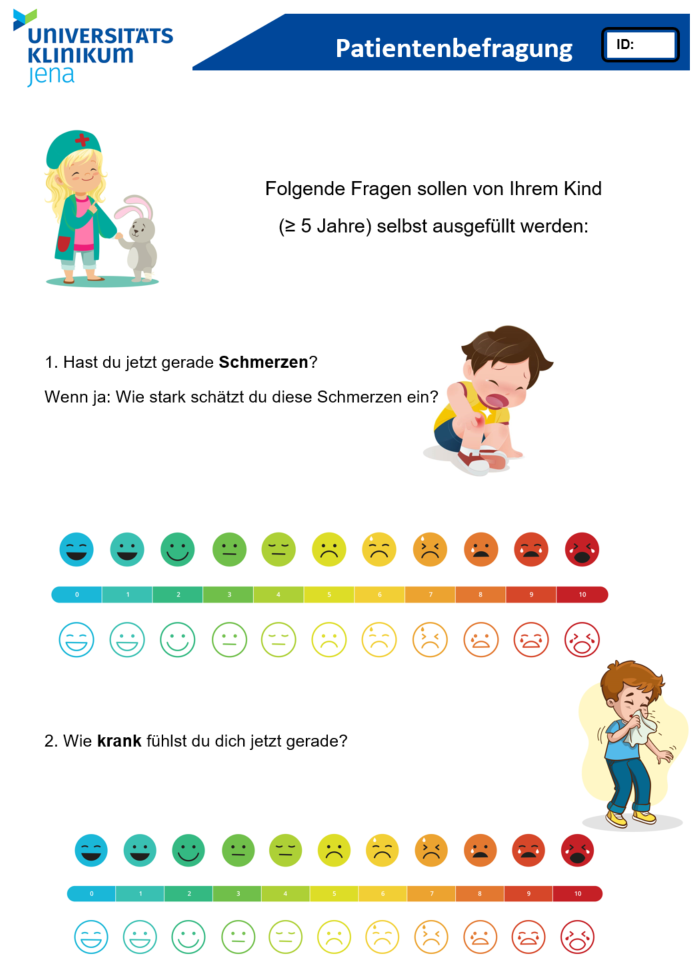 | 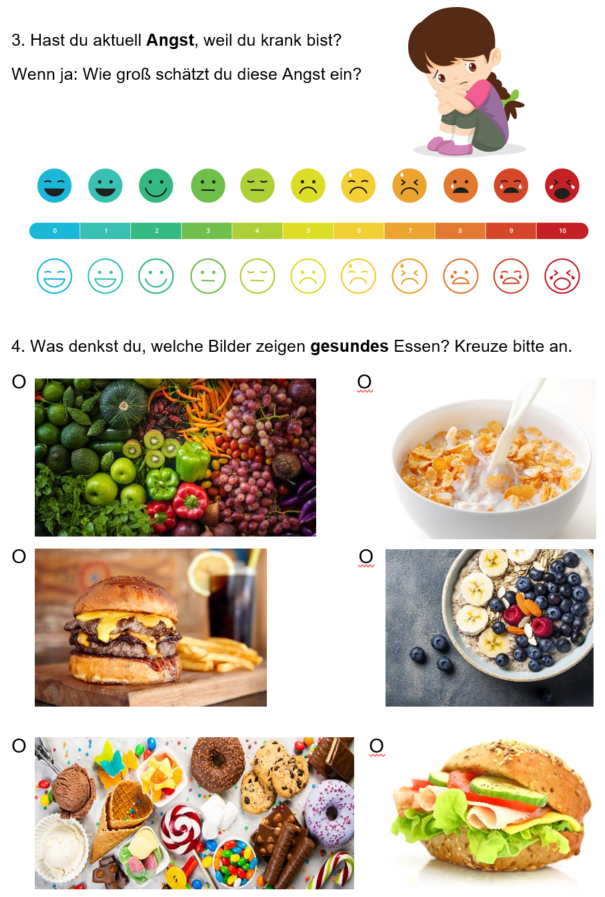 |
| --- | --- |
| *Page 1* | *Page 2*  *(including FL assessment)* |

## Parent questionnaire – additional questions:

“*How long have you been suffering from your current symptoms?”;* less than 24 hours, 24 hours until 2 days, 2 days until one week, 1 week or longer. Additionally, the type of admission (self, family or friends, physician, or employer) was asked.

## HLS-EU-Q16

Consistent with the preceding study[1]. The administration and analysis of HLS-EU-Q16 followed established guidelines and best practices [2, 3]. Permission for its use was obtained. If two or more answers were missing, the questionnaire was deemed incomplete, and this patient’s data excluded from analysis.

The HLS-EU-Q16 is a shortened of the HLS-EU-Q47, which was developed by Sørensen et al. in the Consortium of the European Health Literacy Project in 2009 [2] and first used in the European Health Literacy Survey (HLS-EU) in 2011[4]. It is intended to measure a person's health literacy through self-assessment. All HLS-EU questionnaires are based on the integrated model of health literacy as proposed by Sørensen et al. [5]. The HLS-EU-Q16 was developed inside the Consortium of the European Health Literacy Project using data from the first HLS-EU [6, 7]. In this study, we used the German version of the HLS-EU-Q16 questionnaire, see Table 1.

The 16 items of the HLS-EU-Q16 were selected so that they represent the matrix of the original 47 items as consistently as possible and at the same time exhibit the best possible psychometrics. The selection was made using a single-parameter dichotomous Rasch model and Item Response Theory[6, 8]. They cover all three areas of health literacy that are examined in the HLS-EU-Q47: Health Care (question 1-7), Disease Prevention (question 8-12), and Health Promotion (question 13-16). They also cover all four dimensions of health-related information management: accessing and obtaining information, understanding information, processing information and applying information. Only the ‘use of information’ aspect of ‘health promotion’ is not covered in HLS-EU-Q16 compared to HLS-EU-Q47, as none of the original items met the Rasch criteria. During validation, the correlation of HLS-EU-Q16 results with results from HLS-EU-Q47 were very high (mean *r* = 0.82, range *r* = 0.73-0.88 for different language versions).

In addition to reducing the items, the four possible answers (very easy, rather easy, rather difficult, very difficult) are merged into two categories (easy, difficult) during analysis, resulting in a dichotomous (0, 1) sum score and therefore level allocation: inadequate HL (0-8 points), problematic HL (9-12 points), and adequate HL (13-16 points). Inadequate and problematic HL can be combined as limited HL.

We chose the HLS-EU-Q16 because its short application time of about 3 to 5 minutes makes it well suited for the dynamic environment of an ED. At the same time, the HLS-EU-Q16 is a widely used and generally recognized tool for assessing HL in individuals[9]. Naturally, the results of HLS-EU-Q16 are less robust compared to HLS-EU-Q47: it shows an early ceiling effect for higher HL levels [9], but this is not detrimental for our investigation. Higher HL is not separated into sufficient and excellent HL in the HLS-EU-Q16 but is summarized as adequate HL. While well-validated for general HL assessment, the test may lack sensitivity for nuanced analysis of acute situational judgment in emergency contexts.

| Nr | **Question** | **Domain** |
| --- | --- | --- |
|  | Auf einer Skala von sehr einfach bis sehr schwierig:  Wie einfach ist es Ihrer Meinung nach… |  |
| 1 | …Informationen über Therapien für Krankheiten, die Sie betreffen, zu finden? | **Health Care** |
| 2 | …herauszufinden, wo Sie professionelle Hilfe erhalten, wenn Sie krank sind? |  |
| 3 | …zu verstehen, was Ihr Arzt Ihnen sagt? |  |
| 4 | …die Anweisungen Ihres Arztes oder Apothekers zur Einnahme der verschriebenen Medikamente zu verstehen? |  |
| 5 | …zu beurteilen, wann Sie eine zweite Meinung von einem anderen Arzt einholen sollten? |  |
| 6 | …mit Hilfe der Informationen, die Ihnen der Arzt gibt, Entscheidungen bezüglich Ihrer Krankheit zu treffen? |  |
| 7 | …den Anweisungen Ihres Arztes oder Apothekers zu folgen? |  |
| 8 | …Informationen über Unterstützungsmöglichkeiten bei psychischen Problemen, wie Stress oder Depression, zu finden? | **Disease**  **Prevention** |
| 9 | …Gesundheitswarnungen vor Verhaltensweisen wie Rauchen, wenig Bewegung oder übermäßiges Trinken zu verstehen? |  |
| 10 | …zu verstehen, warum Sie Vorsorgeuntersuchungen brauchen? |  |
| 11 | …zu beurteilen, ob die Informationen über Gesundheitsrisiken in den Medien vertrauenswürdig sind? |  |
| 12 | …aufgrund von Informationen aus den Medien zu entscheiden, wie Sie sich vor Krankheiten schützen können? |  |
| 13 | …Informationen über Verhaltensweisen zu finden, die gut für Ihr psychisches Wohlbefinden sind? | **Health**  **Promotion** |
| 14 | …Gesundheitsratschläge von Familienmitgliedern oder Freunden zu verstehen? |  |
| 15 | …Informationen in den Medien darüber, wie Sie Ihren Gesundheitszustand verbessern können, zu verstehen? |  |
| 16 | …zu beurteilen, welche Alltagsgewohnheiten mit Ihrer Gesundheit zusammenhängen? |  |

Table 1: HLS-EU-Q16 German used in this study [2, 7]

## Categories of diagnoses

| **Diagnosis Severity Category** | **Examples** |
| --- | --- |
| **0**  Not threatening |  |
| **1**  Barely threatening | Finger pain |
| **2**  Possibly threatening | Conjunctivitis |
| **3**  Threatening | Bruise to the forehead |
| **4**  Possibly serious | Tonsillitis |
| **5**  Serious | Purulent otitis media |
| **6**  Very serious | Abscess after insect bite |
| **7**  Critical | Testicular torsion |
| **8**  Likely life-threatening | Facial phlegmon |
| **9**  Life-threatening | Sepsis |
| **10**  Immediately life-threatening | Ketoacidosis |

Table 2: Diagnosis severity assessments by four pediatric emergency medical treatment specialists with example. Only examples that occurred in our study were listed. 0 points were not assigned.

## Diagnoses of included patients

The diagnoses of the included patients were distributed as expected without deviation from common reasons for presentation to the PED. The ICD10 diagnosis groups used for treatment were also used for the analysis. Head laceration and gastroenteritis were the most common (4%), followed by acute upper respiratory tract infection, trauma and fever (2-3%). Separated by the three HL levels, this distribution did not change significantly. The overall distribution of diagnoses in the included patients was representative of the common reasons for presentation to our PED. The upper quantile in terms of severity assessment by specialists comprised 36 patients, one of whom died during inpatient treatment. 28 patients from this group were included in the analysis as Severe Outcome (distribution-based and based on clinical course), including the deceased patient. For the clinically defined severe outcome the most frequently met criterion was inpatient admission for more than 24 hours (90%), care escalation within 24 hours (36%), and/or surgical procedure or invasive intervention within 72 hours (32%). Distribution did not differ by HL group (p > .05). The selected clinical markers (vital sign monitoring >12 hours, inpatient admission >24 hours, care escalation within 24 hours, ICU admission, parenteral antibiotics, and surgical/invasive intervention ≤72 hours) represent established indicators of adverse outcomes in pediatric emergency cohorts, reflecting clinical acuity beyond initial triage. This composite aligns with recommended endpoints for severe pediatric illness[10, 11] and maintains study feasibility without invasive data.

## Sensitivity analyses of Discrepancy thresholds

To assess robustness of the Discrepancy operationalization, we conducted sensitivity analyses using three Concordance thresholds on signed, covariate-adjusted discrepancy scores: baseline (−1 to +1), stricter (−0.5 to +0.5), and more lenient (−2 to +2). Associations between parental HL level and Discrepancy category (Negative, Concordance, Positive) remained non-significant (p > .05) across all thresholds for most pairs, confirming no systematic HL effect. The core finding - that underestimation relative to specialists predicts higher Severe Outcome rates, while overestimation predicts lower rates - was robust across thresholds.

## Study Sample Size:

The sample size was based on the precision (95% confidence interval length) of the correlation between the endpoint emergency condition severity, separately assessed by patient, parent, nurse, and physician. In the pilot and preceding trial, a moderate correlation (0.30) was found, requiring the analysis of 180 patients to be ensure the confidence interval does not exceed 0.3. Sample size for the main study was subsequently powered primarily for estimation of inter-rater correlations (target CI width ≤0.3 around ρ=0.30), therefore developmental/self-report effects were more relevant than HL gradients. Accounting for a 10% dropout rate, 200 patients had to be recruited.

## Biometrical Approach

Descriptive statistics (mean, SD, n) summarized all continuous variables. Distributions were evaluated using Shapiro–Wilk tests and Q–Q plots; mild deviations from normality led to the use of non-parametric tests for group comparisons. Spearman rank-order correlations (ρ) quantified the strength and direction of agreement between rater groups (parents, patients, nurses, physicians, and specialists). Fisher’s z values indexed effect size and allowed interpretation of correlation magnitude. For discrepancy analyses, signed and absolute discrepancy scores were computed between family (parent, patient) and professional (team, specialist) assessments. Signed scores reflected direction (negative = underestimation, positive = overestimation), whereas absolute scores indicated the magnitude of disagreement. Group comparisons across the three HL categories (*adequate*, *problematic*, *inadequate*) were performed using the Kruskal–Wallis test, followed by Dunn post-hoc contrasts with Holm-corrected p values. Effect sizes were expressed as eta-squared (η²). Adjusted ratings were computed using OLS residualization to control for demographic covariates: parental ratings were adjusted for parent age, education, and gender, while patient ratings were adjusted for patient age. Discrepancy classifications (Negative, Concordance, Positive) were based on signed adjusted values (< −1, −1–1, > 1). The association between discrepancy direction and severe clinical outcome was examined overall and across HL groups using chi-square tests of independence. The Severe Outcome variable was defined distributionally as a specialist’s rating ≥ 1 SD above the mean (cutoff = 5.70 on the adjusted scale, or 5.80 for non-adjusted ratings, i.e. 6 on the Likert-Scale). Logistic regression analyses (non-adjusted ratings) predicted this outcome from (a) individual rater assessments (parents, patients, nurses, physicians) and (b) combined multivariable models including all four raters simultaneously. Models reported unstandardized B, SE, Wald χ², p, odds ratios (OR) with 95% confidence intervals, and McFadden’s R² as effect size. Additional models examined age interactions (e.g., *Age × Anxiety*) to test age-dependent validity of child self-reports. Unstandardized coefficients (B), t values, R², and two-tailed p values (α = .05) are reported.

## Covariate Adjustment Procedure:

Severity assessments were covariate-adjusted with a regression-based revisualization procedure. Separate Ordinary Least Squares models were estimated for each assessment source. Parents’, nurses’, physicians’, and specialists’ assessments were adjusted for parental age, parental education, and parental gender, whereas patients’ assessments were adjusted only for child age, reflecting the distinct sources of demographic influence on each rating. All covariates were centered on their grand means before estimation. For each case, the covariate-related component of the prediction was removed from the raw rating, and the overall mean of the rating was added back. This yielded an adjusted value representing the assessment that would have been observed if all covariates had been at their sample-average levels.

## Agreement metrics

Quadratically weighted κ confirmed moderate professional–specialist agreement and weak lay–professional agreement, see *Table 3*. These metrics align with Spearman results, showing fair-to-moderate professional concordance and lower lay-professional overlap.

|  | **Weighted κ [95% CI]** |
| --- | --- |
| Nurse–Specialists | 0.33 [0.19, 0.47] |
| Physician–Specialists | 0.22 [0.08, 0.36] |
| Parent–Specialists | 0.08 [-0.03, 0.20] |
| Patient–Specialists | 0.03 [-0.11, 0.16] |
| Parent–Medical Team | 0.20 [0.09, 0.30] |
| Patient–Medical Team | 0.08 [-0.08, 0.24] |

Table 3: Quadratically weighted κ representing agreement between assessments.

## HL Covariate collinearity analysis

| **Covariate** | **ρ [95% CI]** | **p** |
| --- | --- | --- |
| University Education (yes) | -0.01 [-0.15, 0.14] | .900 |
| Parent Age | -0.05 [-0.2, 0.09] | .468 |
| Education (ordinal 1–4) | 0.04 [-0.12, 0.18] | .634 |

*Table 4: Spearman’s correlation with continuous HL sum score (HLS-EU-Q16, 0-16).*
